# Multi-modal AI in precision medicine: integrating genomics, imaging, and EHR data for clinical insights

**DOI:** 10.3389/frai.2025.1743921

**Published:** 2026-01-07

**Authors:** Shahper Nazeer Khan, Danishuddin, Mohd Wajid Ali Khan, Luca Guarnera, Syed Mohammad Fauzan Akhtar

**Affiliations:** 1 Integral Centre of Excellence for Interdisciplinary Research (ICEIR), Integral University, Lucknow, India; 2 Department of Biosciences, Integral University, Lucknow, India; 3 Department of Biotechnology, Yeungnam University, Gyeongsan, Republic of Korea; 4 Department of Chemistry, College of Sciences, University of Hail, Hail, Saudi Arabia; 5 Medical and Diagnostic Research Center, University of Hail, Hail, Saudi Arabia; 6 Policlinico Tor Vergata, Rome, Italy; 7 Integral Institute of Medical Science and Research, Integral University, Lucknow, India

**Keywords:** artificial intelligence, multi-modal integration, data-driven medicine, personalized treatment, AI-driven clinical decision, healthcare, public health

## Abstract

Precision healthcare is increasingly oriented toward the development of therapeutic strategies that are as individualized as the patients receiving them. Central to this paradigm shift is artificial intelligence (AI)-enabled multi-modal data integration, which consolidates heterogeneous data streams—including genomic, transcriptomic, proteomic, imaging, environmental, and electronic health record (EHR) data into a unified analytical framework. This integrative approach enhances early disease detection, facilitates the discovery of clinically actionable biomarkers, and accelerates rational drug development, with particularly significant implications for oncology, neurology, and cardiovascular medicine. Advanced machine learning (ML) and deep learning (DL) algorithms are capable of extracting complex, non-linear associations across data modalities, thereby improving diagnostic precision, enabling robust risk stratification, and informing patient-specific therapeutic interventions. Furthermore, AI-driven applications in digital health, such as wearable biosensors and real-time physiological monitoring, allow for continuous, dynamic refinement of treatment plans. This review examines the transformative potential of multi-modal AI in precision medicine, with emphasis on its role in multi-omics data integration, predictive modeling, and clinical decision support. In parallel, it critically evaluates prevailing challenges, including data interoperability, algorithmic bias, and ethical considerations surrounding patient privacy. The synergistic convergence of AI and multi-modal data represents not merely a technological innovation but a fundamental redefinition of individualized healthcare delivery.

## Introduction

Personalized medicine, also known as precision medicine, is a revolutionary approach to healthcare that tailors medical treatment to the individual characteristics of each patient. Unlike traditional medical practices, which adopt a “one-size-fits-all” strategy, personalized medicine leverages genetic, environmental, and lifestyle factors to optimize disease prevention, diagnosis, and therapy. The advent of high-throughput sequencing, molecular diagnostics, and computational biology has significantly advanced the field, allowing for more precise interventions. Personalized medicine holds the potential to enhance treatment efficacy while minimizing adverse effects, thereby improving patient outcomes and reducing healthcare costs (Khan et al., 2025; García, 2024). The growing accessibility of multi-omics data including genomics, transcriptomics, proteomics, and metabolomics has further propelled the development of personalized medicine. By integrating these diverse datasets, clinicians can better predict disease susceptibility, identify optimal treatment strategies, and monitor therapeutic responses. This paradigm shift in medicine underscores the necessity of advanced computational tools to manage and interpret complex biological data, paving the way for artificial intelligence (AI) to play a transformative role (Bahmani et al., 2025; Topol, 2018; Li et al., 2017; Hood and Friend, 2011). AI is redefining healthcare by enabling more precise, efficient, and predictive patient-specific treatments. Through machine learning (ML) algorithms, deep learning models, and natural language processing (NLP), AI can process vast amounts of biomedical data, uncover hidden patterns, and generate actionable insights for clinicians. AI-driven analytics assist in disease risk assessment, early diagnosis, drug discovery, and therapeutic optimization (He et al., 2025).

One of AI’s most profound impacts is in oncology, where it helps stratify patients based on genetic mutations and molecular markers, leading to more targeted therapies. AI-powered imaging techniques enhance early cancer detection, while predictive models refine treatment regimens by analyzing individual patient responses. Additionally, AI facilitates the identification of rare diseases by recognizing complex phenotypic and genotypic correlations that may elude conventional diagnostic methods (Bhattacharya et al., 2024; Ching et al., 2018). Beyond diagnostics and treatment, AI is instrumental in advancing pharmacogenomics—the study of how genetic variations influence drug responses. By integrating AI with pharmacogenomic data, clinicians can personalize drug prescriptions, mitigating adverse drug reactions and optimizing therapeutic efficacy. Furthermore, AI-driven robotic surgery and virtual health assistants improve patient engagement and accessibility to personalized medical advice, contributing to a more patient-centric healthcare system (Bajwa et al., 2021). The convergence of AI, big data, and bioinformatics is transforming healthcare into a highly data-driven domain. The exponential growth of healthcare data, including electronic health records (EHRs), medical imaging, wearable sensor data, and multi-omics profiles, necessitates robust computational frameworks to extract meaningful insights. AI, when integrated with big data analytics, facilitates real-time decision-making, predictive modeling, and precision diagnostics (Si et al., 2020; Obermeyer and Emanuel, 2016).

Bioinformatics serves as the backbone of AI-driven personalized medicine by enabling the systematic analysis of complex biological datasets. AI-powered bioinformatics tools can annotate genomic sequences, predict protein structures, and analyze gene expression patterns to elucidate disease mechanisms. The integration of AI with network medicine further enhances our understanding of intricate biological interactions, fostering the development of novel therapeutic strategies (Abdelhalim et al., 2022; Greener et al., 2021). AI-driven population health management is another critical advancement, leveraging predictive analytics to identify at-risk populations, track disease outbreaks, and optimize public health interventions. AI also aids in clinical trials by accelerating patient recruitment, monitoring treatment responses, and identifying novel drug targets, thereby expediting the drug development process (Lu et al., 2024; Kaur and Butt, 2025). Despite its transformative potential, AI-driven personalized medicine faces challenges, including ethical considerations, data privacy concerns, and the need for regulatory frameworks to ensure algorithmic transparency and reliability. Addressing these challenges will be crucial in realizing the full potential of AI in revolutionizing healthcare (Morley et al., 2019; Rajkomar et al., 2018).

### The evolution of personalized medicine

The transition from a one-size-fits-all approach to tailored treatments marks a significant milestone in the evolution of healthcare. Historically, medical treatments were designed based on broad population-level data, often neglecting the genetic and environmental variability among individuals (Collins and Varmus, 2015). However, advancements in genomics and biotechnology have ushered in an era where therapies are customized to the molecular and genetic profiles of patients (Khan et al., 2025; García, 2024). Key breakthroughs in genomics, particularly the completion of the Human Genome Project in 2003, have provided a foundation for personalized medicine. This project enabled the identification of genetic variations associated with various diseases, paving the way for targeted therapies. The emergence of next-generation sequencing (NGS) has further accelerated the ability to decode individual genomes, allowing for precise disease risk assessment and tailored interventions (Denny and Collins, 2021; Green et al., 2020). Biotechnology has played a crucial role in the development of personalized medicine. The advent of CRISPR-based gene editing has opened new possibilities for correcting genetic mutations responsible for inherited disorders. Additionally, advancements in single-cell sequencing and proteomics have facilitated a deeper understanding of disease mechanisms, enhancing the potential for personalized therapeutic strategies (Jinek et al., 2012; Lu et al., 2024). The integration of AI into personalized medicine has been transformative, particularly in processing vast and complex biological datasets. AI-driven algorithms can analyze genomic, clinical, and imaging data to identify disease subtypes and predict patient responses to specific treatments. AI applications in drug discovery have also revolutionized the identification of novel therapeutic targets, significantly reducing the time and cost associated with drug development (Zhavoronkov, 2018; He et al., 2025). Several case studies highlight the impact of AI on precision medicine. For instance, IBM Watson for Oncology has demonstrated success in analyzing patient records and recommending tailored cancer treatments based on vast biomedical literature. Similarly, AI-powered predictive models have been used in cardiovascular medicine to assess individual risk factors and recommend personalized interventions (Singh et al., 2024; Lüscher et al., 2024). In oncology, AI-driven molecular profiling has been instrumental in identifying targeted therapies for patients with specific genetic mutations. The use of AI in analyzing liquid biopsies has enabled non-invasive cancer detection, improving early diagnosis and treatment planning (Foser et al., 2024; Topol, 2018). The evolution of personalized medicine continues to accelerate, driven by the convergence of genomics, biotechnology, and AI. As these technologies advance, the potential for more precise, efficient, and patient-centric healthcare solutions becomes increasingly evident.

### Role of AI in personalized medicine

The integration of artificial intelligence (AI) in personalized medicine is revolutionizing the landscape of healthcare, offering unprecedented potential for the optimization of patient care. Personalized medicine tailors medical treatment to individual characteristics, such as genetic profiles, lifestyle, and environmental factors. AI, particularly machine learning (ML) and deep learning (DL), plays a pivotal role in advancing personalized medicine by enhancing early disease detection, improving drug development, and refining treatment plans for patients.

#### Machine learning for disease prediction and diagnosis

One of the most promising applications of AI in personalized medicine is in the early detection and diagnosis of diseases. Machine learning algorithms have proven to be highly effective in recognizing patterns within complex datasets, allowing for earlier diagnosis of conditions such as cancer, diabetes, and cardiovascular diseases, often before clinical symptoms manifest. For example, AI models have been developed to analyze medical imaging, such as mammograms and CT scans, for signs of malignancies with high accuracy (Esteva et al., 2017). These models use vast datasets of labeled images to “learn” the features indicative of early-stage cancers, aiding radiologists in identifying potential issues faster than traditional methods. AI’s ability to analyze genomic and clinical data also extends to predictive analytics in assessing patient risk for various diseases. By integrating patient history, lifestyle data, and genetic information, AI algorithms can forecast the likelihood of a disease developing, enabling proactive intervention. For instance, AI models in cardiology can predict the risk of heart attacks by analyzing historical patient data, including blood pressure, cholesterol levels, and genetic markers, facilitating early intervention to reduce mortality rates (Rajkomar et al., 2018). In diabetes management, ML models can predict the onset of complications such as retinopathy or nephropathy, based on patterns in longitudinal patient data, allowing clinicians to tailor preventative measures for high-risk individuals (Khokhar et al., 2025).

#### AI-driven drug development and pharmacogenomics

AI’s role in drug development and pharmacogenomics has garnered significant attention, particularly for its ability to expedite the discovery of novel therapeutics and optimize drug use based on individual genetic profiles. Traditional drug development processes are often slow and costly, with many candidates failing in clinical trials due to unforeseen adverse effects or lack of efficacy. However, AI has the potential to streamline this process by predicting the interaction of compounds with target proteins and identifying viable drug candidates more efficiently. For example, deep learning techniques have been employed to predict the binding affinity between small molecules and proteins, facilitating virtual screening of chemical libraries (Gawehn et al., 2015). This AI-driven approach not only accelerates drug discovery but also enables drug repurposing, identifying existing drugs that may be effective against new or underexplored diseases (Askr et al., 2022). In the realm of pharmacogenomics, AI models are increasingly being used to understand how genetic variations influence individual drug responses. By analyzing genetic data from large patient cohorts, AI algorithms can identify biomarkers that predict how patients will respond to specific drugs, thereby enabling more targeted therapies. For instance, AI has been applied to pharmacogenomic databases to uncover genetic variants associated with adverse drug reactions, which could lead to safer, more personalized prescriptions (He et al., 2025). This data-driven approach also holds promise for optimizing the dosage of medications to avoid toxicity or insufficient therapeutic effects, particularly for drugs with a narrow therapeutic index.

#### Personalized treatment planning with AI

AI’s contribution to personalized treatment planning is perhaps one of the most transformative aspects of its integration into medicine. By leveraging patient-specific data, including genetic information, lifestyle factors, and clinical history, AI-powered systems can assist clinicians in formulating individualized treatment plans. Deep learning algorithms, for example, can analyze complex patient datasets to provide personalized predictions regarding the best course of treatment, taking into account factors such as treatment efficacy, potential side effects, and the patient’s overall health status. In oncology, AI has been utilized to guide treatment decisions by analyzing genomic alterations in tumors and suggesting targeted therapies that have the highest likelihood of success based on molecular profiling (Luchini et al., 2021; Lotter et al., 2024). AI-assisted decision-making tools are also enhancing clinical workflows by providing evidence-based recommendations for drug combinations or interventions tailored to individual patients. These systems reduce the cognitive load on healthcare providers, allowing for quicker and more accurate decision-making, especially in fast-paced environments like emergency departments. Moreover, AI-driven decision support systems can incorporate real-time data from wearable health devices, allowing for continuous monitoring of a patient’s condition and dynamic adjustment of treatment plans as new data becomes available. The application of deep learning in medical imaging is another significant advancement in personalized treatment planning. AI models are now routinely used to analyze medical images with high precision, enabling earlier detection of abnormalities and aiding in the assessment of disease progression. For instance, AI algorithms trained on large datasets of radiological images can detect subtle signs of disease that might be missed by the human eye, improving diagnostic accuracy. In conditions such as brain tumors, AI can also predict patient prognosis based on the imaging characteristics of the tumor, helping clinicians to tailor treatment plans more effectively (Li et al., 2023a).

### Data sources and AI models in personalized medicine

**Figure 1:**
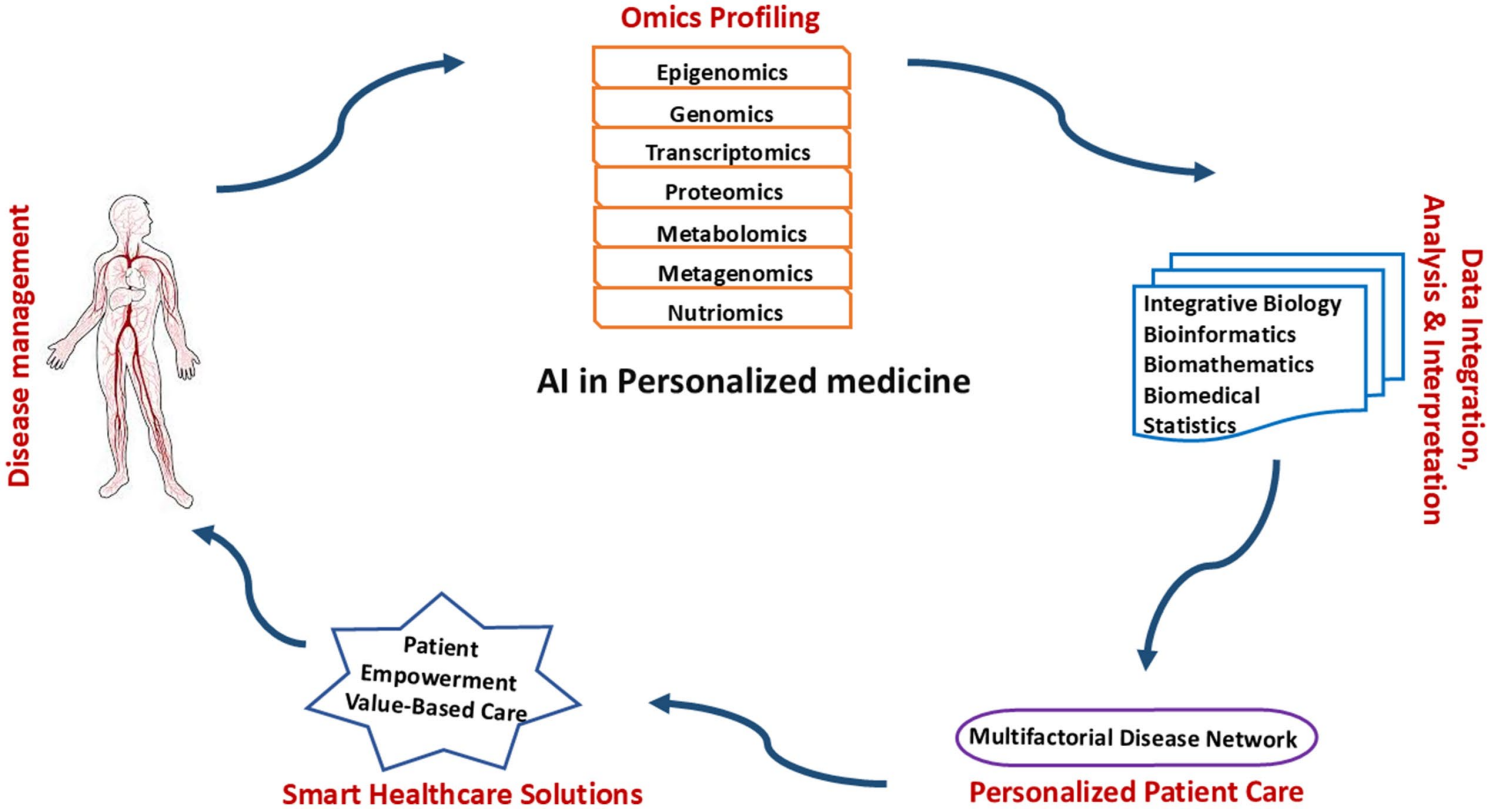
A schematic representation showing how AI integrates multi-omics data, electronic health records (EHRs), and medical imaging to provide personalized treatment.

The integration of artificial intelligence (AI) with diverse biomedical data sources is a cornerstone of modern personalized medicine. AI-driven approaches leverage vast and complex datasets to generate predictive insights, enhance diagnostics, and tailor individualized therapeutic strategies. The efficacy of AI models in personalized medicine relies on their ability to process and analyze data from multiple sources, including genomic and multi-omics datasets, electronic health records (EHRs), and real-time patient monitoring systems. A schematic representation showing how AI integrates multi-omics data, electronic health records (EHRs), and medical imaging to provide personalized treatment (Figure 1).

#### Genomic and multi-omics data integration

The advent of high-throughput sequencing technologies has revolutionized biomedical research by generating vast amounts of genomic and multi-omics data, including transcriptomics, proteomics, epigenomics, and metabolomics (Hasin et al., 2017). AI models, particularly deep learning, play a pivotal role in integrating and analyzing these multi-dimensional datasets to uncover molecular mechanisms underlying disease susceptibility and drug responses (Angermueller et al., 2016). Genomic data alone provides valuable insights into genetic predispositions and disease risk, but multi-omics integration enhances predictive accuracy by considering post-translational modifications, gene–environment interactions, and dynamic regulatory networks (Molla and Bitew, 2024). AI algorithms can identify biomarkers and therapeutic targets with high precision, facilitating the development of personalized treatment plans. For example, convolutional neural networks (CNNs) and recurrent neural networks (RNNs) have been used to predict the impact of genetic variants on protein function and drug metabolism, leading to improved pharmacogenomic interventions (Chang et al., 2025).

#### Electronic health records (EHRs) and AI-driven insights

EHRs serve as a rich source of patient data, encompassing demographics, clinical histories, laboratory results, imaging data, and physician notes. AI-driven analytics transform EHRs into powerful tools for precision medicine by identifying disease patterns, optimizing treatment plans, and predicting patient outcomes (Rajkomar et al., 2018). Machine learning algorithms, including gradient boosting and random forests, have been employed to predict disease progression by analyzing structured and unstructured EHR data. One of the key challenges in leveraging EHRs is data heterogeneity and missing information. AI models employing imputation techniques, such as generative adversarial networks (GANs), can address these limitations by reconstructing incomplete datasets with high fidelity (Nogues et al., 2024). Additionally, NLP has been instrumental in extracting valuable insights from unstructured clinical notes, enhancing disease classification and enabling personalized risk assessment (Scharp et al., 2023).

#### AI techniques: deep learning, reinforcement learning, and NLP

The implementation of AI in personalized medicine relies on several advanced techniques, including deep learning, reinforcement learning, and NLP. Deep learning, particularly CNNs and long short-term memory (LSTM) networks, has demonstrated remarkable success in analyzing complex biomedical data. CNNs have been widely applied in medical imaging to detect early-stage diseases, while LSTMs are used in genomic sequence analysis and predictive modeling of disease trajectories (Litjens et al., 2017). Whereas reinforcement Learning approach enables AI models to optimize treatment strategies dynamically based on patient responses. In oncology, reinforcement learning algorithms have been employed to personalize chemotherapy regimens, balancing efficacy and toxicity (Frommeyer et al., 2025). In natural Language Processing (NLP) techniques, such as Bidirectional Encoder Representations from Transformers (BERT) and GPT-based models, enhance clinical decision-making by processing and interpreting unstructured medical texts, pathology reports, and scientific literature (Lee et al., 2019). These models facilitate automated disease classification and patient stratification, further advancing precision medicine.

### AI applications in key areas of personalized medicine

The advent of artificial intelligence (AI) in personalized medicine has revolutionized patient-specific diagnostics, prognostics, and therapeutic decision-making. AI models integrate vast and complex biomedical datasets including multi-omics profiles, electronic health records (EHRs), and medical imaging data to uncover disease signatures and optimize patient management strategies. AI’s transformative role is particularly evident in oncology, neurology, cardiovascular medicine, and the diagnosis of rare genetic disorders, where precision and early intervention are crucial.

#### Oncology: AI-guided cancer treatment and immunotherapy

**Figure 2:**
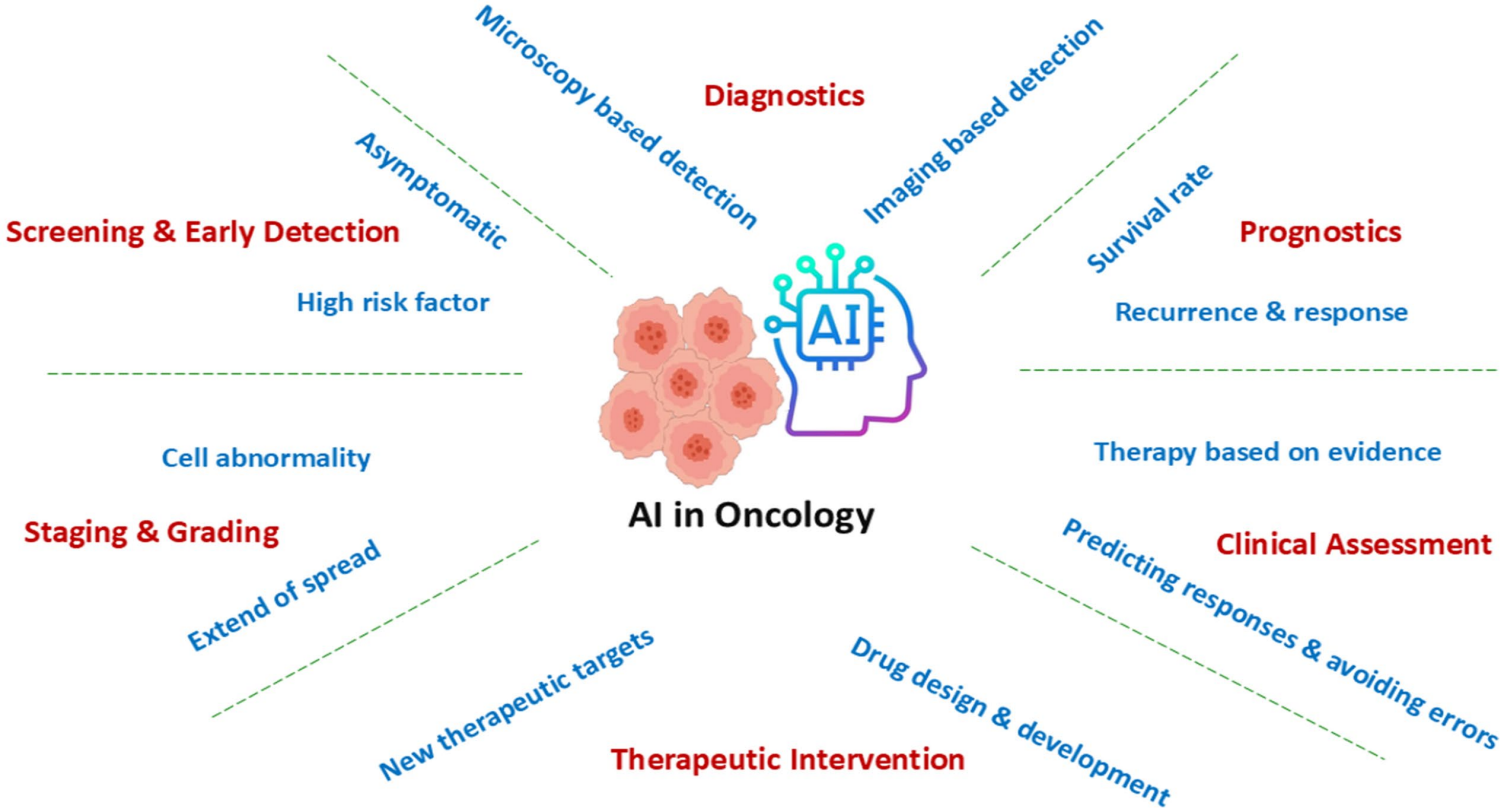
AI-assisted cancer management and its role in different phases of disease.

AI is reshaping cancer treatment paradigms through the integration of machine learning (ML) and deep learning (DL) models with genomic and imaging datasets (Li et al., 2023b). Convolutional neural networks (CNNs) and transformer-based architectures such as Vision Transformers (ViTs) have demonstrated superior accuracy in analyzing radiological and histopathological images to detect malignant lesions at early stages (Ardila et al., 2019). AI-enhanced liquid biopsy analysis enables the detection of circulating tumor DNA (ctDNA) and tumor-derived exosomes, facilitating non-invasive early cancer screening (Hussain et al., 2025). AI-driven multi-omics integration allows for the precise identification of molecular subtypes in aggressive cancers such as triple-negative breast cancer (TNBC) and glioblastoma (Ballard et al., 2024). Furthermore, deep reinforcement learning (DRL) algorithms optimize treatment regimens by continuously adapting therapeutic strategies based on real-time tumor response data (Yang et al., 2022). In immunotherapy, AI models leverage transcriptomic and single-cell sequencing data to predict patient responsiveness to immune checkpoint inhibitors (ICIs) by identifying tumor microenvironment immune signatures (Li et al., 2023b). Hence, AI-assisted cancer management is aiding the risk stratification and early detection, through diagnosis, treatment planning, and real-time monitoring, to survivorship and end-of-life care—enabling precision interventions, personalized prognoses, and improved quality of life (Figure 2).

#### Neurology: AI in Alzheimer’s and Parkinson’s disease management

AI has significantly advanced early diagnosis and progression modeling of neurodegenerative diseases such as Alzheimer’s disease (AD) and Parkinson’s disease (PD). Graph-based deep learning architectures have been applied to neuroimaging modalities, including amyloid PET and functional MRI, to detect subtle structural and functional alterations indicative of preclinical AD (Bazarbekov et al., 2024). Recurrent neural networks (RNNs) trained on longitudinal EHRs predict cognitive decline trajectories, enabling early therapeutic interventions (Jasodanand et al., 2025). In PD, AI-powered wearable biosensors capture gait, tremor, and speech abnormalities, offering real-time, objective disease monitoring (Sakar et al., 2018). Generative adversarial networks (GANs) reconstruct high-resolution imaging data from low-quality scans, enhancing the accuracy of PD-related dopamine transporter imaging (Twala et al., 2025). Moreover, integrative AI models analyzing genomic, proteomic, and gut microbiome data have identified novel PD biomarkers, paving the way for personalized neuroprotective therapies.

#### Cardiovascular health: AI for heart disease risk prediction

AI-driven predictive analytics play a crucial role in cardiovascular medicine, enabling early detection and personalized management of heart disease (He et al., 2018). Transformer-based architectures, such as bidirectional encoder representations from transformers (BERT), analyze vast clinical datasets to predict the onset of heart failure and atrial fibrillation with high accuracy (Biondi-Zoccai et al., 2025). Deep learning-assisted echocardiographic analysis automates left ventricular ejection fraction (LVEF) quantification, improving heart failure diagnosis (Asch et al., 2019). Genomic risk prediction models powered by AI facilitate stratification of individuals based on polygenic risk scores (PRS) for coronary artery disease (CAD) and myocardial infarction (Xie et al., 2023). AI-assisted computational modeling of cardiac electrophysiology supports patient-specific treatment planning, guiding optimal antiarrhythmic therapy and catheter ablation strategies (Attia et al., 2019).

#### Rare diseases: AI-enabled genetic analysis for better diagnosis

Rare genetic diseases often present diagnostic challenges due to heterogeneous phenotypic expressions and limited available patient data. AI models trained on large-scale genomic databases enable rapid prioritization of pathogenic variants in whole-genome and whole-exome sequencing (Kafkas et al., 2025). AI-driven knowledge graphs integrate multi-omics and phenotypic data, facilitating automated disease classification and novel disease-gene association discovery (Chandak et al., 2023). Deep learning-based facial recognition technology aids in the diagnosis of syndromic genetic disorders by analyzing craniofacial phenotypes from patient images (Gurovich et al., 2018). Additionally, natural language processing (NLP) algorithms extract disease-specific information from unstructured clinical notes, accelerating differential diagnosis and clinical decision-making for rare condition.

### Ethical, legal, and social challenges

AI-driven healthcare systems rely heavily on large datasets containing sensitive patient information, raising significant ethical and legal concerns (Bouderhem, 2024). Ensuring data privacy while maintaining accessibility for research and clinical use remains a major challenge. Regulatory frameworks such as the General Data Protection Regulation (GDPR) in Europe, the Health Insurance Portability and Accountability Act (HIPAA) in the United States, and the Personal Information Protection and Electronic Documents Act (PIPEDA) in Canada provide guidelines on data handling, patient consent, and security protocols (Shabani et al., 2021). A critical concern is informed consent, as AI models require continuous data collection, often from unsuspecting individuals (Chau et al., 2025). Implementing clear and understandable consent mechanisms is essential. Additionally, while data anonymization techniques help protect patient privacy, the risk of re-identification remains, particularly when datasets are cross-referenced (Shojaei et al., 2025). Cyber threats further complicate the landscape, necessitating compliance with stringent security standards such as encryption and secure cloud storage (Bertl et al., 2024). Furthermore, regulatory adaptability is crucial, as existing policies often lag behind rapid AI advancements, requiring flexible mechanisms that evolve alongside new developments (Mennella et al., 2024).

Bias in AI models presents another ethical challenge, particularly in healthcare, where biased predictions can exacerbate health disparities (Obermeyer et al., 2019). Biases often arise from training data limitations, algorithmic biases, and implicit biases in model deployment (Chen et al., 2023; Mehrabi et al., 2021). AI models trained on non-representative datasets may fail to generalize across diverse populations, leading to misdiagnosis or suboptimal treatment recommendations (Hasanzadeh et al., 2025). Some risk assessment tools systematically underrepresent marginalized populations, reinforcing existing social and racial disparities (Obermeyer et al., 2019). Historical biases embedded in healthcare records can also be perpetuated by AI systems, underscoring the need for proactive bias mitigation strategies (Hasanzadeh et al., 2025). One approach to mitigating bias is ensuring diverse data representation by expanding datasets to include underrepresented groups (Hasanzadeh et al., 2025). Algorithmic transparency through open-source AI models and explainable AI (XAI) techniques can help identify and correct biases (Maniatis, 2025). Regulatory oversight, including standardizing AI fairness assessments and incorporating equity audits, is essential for mitigating systemic biases and ensuring equitable healthcare delivery (Rajkomar et al., 2018). Balancing AI automation with human expertise is essential to maintaining ethical and safe healthcare practices (Mosqueira-Rey et al., 2022). While AI can automate diagnostics, streamline administrative tasks, and improve patient outcomes, over-reliance on AI poses risks (Khosravi et al., 2024). Excessive trust in AI predictions may lead clinicians to overlook contextual patient factors. AI should complement rather than replace human judgment, particularly in high-risk scenarios where accountability is crucial (Weiner et al., 2025). Many AI models function as “black boxes,” making it difficult for clinicians to understand their decision-making process, underscoring the importance of explainable AI methods (Pfeifer et al., 2025). A collaborative approach, incorporating human-in-the-loop systems, ensures that AI remains an assistive tool rather than a replacement for healthcare professionals (Mosqueira-Rey et al., 2022). Training clinicians in AI ethics and interpretability equips them to make informed decisions (Topol, 2018). Additionally, continuous AI audits are necessary to assess performance in real-world clinical settings, identify risks, and adjust deployment strategies accordingly (Hassan et al., 2024).

### Future prospects and innovations

Artificial intelligence (AI) is at the forefront of revolutionizing personalized healthcare, offering transformative capabilities in diagnostics, treatment optimization, and drug discovery. Emerging technologies, including quantum computing and AI-driven digital twins, are poised to further accelerate advancements in precision medicine. Additionally, industry-academia collaborations are essential to translating AI innovations into clinically viable solutions, ensuring scalability and regulatory compliance.

#### AI in personalized healthcare

**Figure 3:**
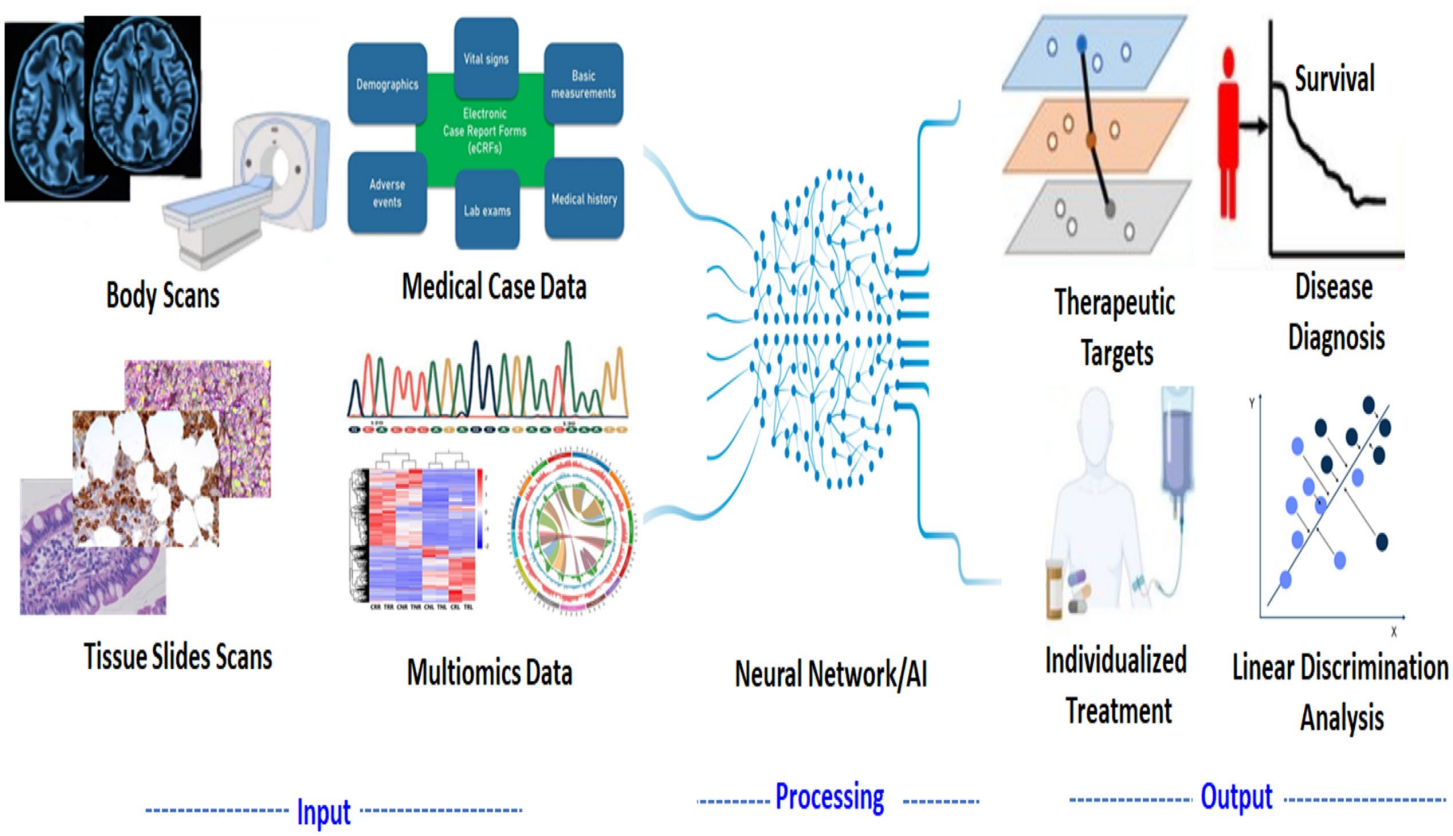
AI-driven workflow for precision medicine: from feature collection to prediction model.

AI-driven tools leverage machine learning (ML), deep learning (DL), and natural language processing (NLP) to analyze multi-modal data, including genomic sequences, electronic health records (EHRs), and medical imaging. This enables early disease detection, risk stratification, and optimized therapeutic interventions. Machine learning models trained on omics data have significantly improved disease prediction and early diagnosis, particularly in oncology, neurology, and cardiology. Deep convolutional neural networks (CNNs) are enhancing radiology and pathology assessments, allowing precise identification of malignant lesions. AI-powered predictive analytics integrating genetic predisposition and lifestyle factors further refine risk assessments and preventive strategies. AI has also transformed drug discovery by accelerating target identification and optimizing compound screening. Deep generative models, including variational autoencoders (VAEs) and reinforcement learning, have successfully identified novel therapeutics. Moreover, AI-driven drug repurposing approaches have uncovered promising candidates for treating rare and emerging diseases. Additionally, personalized treatment regimens tailored to an individual’s genetic, metabolic, and microbiome profiles are being developed using AI-powered decision support systems (Figure 3; Table 1).

**Table 1:** Comprehensive list of AI tools in healthcare.

Category	AI tool	Purpose	Year of launch
Diagnosis and imaging	BM Watson for Oncology	Assists oncologists in treatment recommendations based on clinical evidence.	2013
	Google DeepMind Health (now Google Health AI)	AI for disease detection, especially in radiology and ophthalmology.	2016
	Zebra Medical Vision	AI-powered medical imaging analysis (X-rays, CT scans, MRIs).	2014
	Qure.ai	AI-based interpretation of chest X-rays and brain CT scans.	2016
	*Viz.*ai	AI-driven stroke detection via brain imaging.	2018
	PathAI	AI-assisted pathology for cancer diagnosis.	2017
	Arterys	AI for cardiac imaging and radiology interpretation.	2016
	Aidoc	AI triage system for radiology scans to detect critical conditions.	2016
	Lunit INSIGHT	AI for cancer detection in chest X-rays, mammography.	2019
	RapidAI	AI stroke and neurovascular imaging workflow solution.	2020
	Samsung AI-CT Portfolio (CereTom Elite, OmniTom Elite PCD, BodyTom)	Mobile CT scanners with AI-enabled imaging for ICU, ER, OR use.	2025
	IntelliCardiac	Deep learning platform for 4D cardiac image segmentation and disease classification	2025
Drug discovery and research	Atomwise	AI-driven drug discovery using deep learning.	2012
	BenevolentAI	AI-powered drug development and biomedical research.	2013
	Insilico Medicine	AI for identifying new drug candidates and aging research.	2014
	Exscientia	AI-based drug design and precision medicine.	2012
	BioXcel Therapeutics	AI for repurposing existing drugs for new diseases.	2017
	Recursion Pharmaceuticals	AI + automation for phenomics-driven drug discovery.	2013
	Valo Health	End-to-end AI-driven drug development platform.	2019
	OpenEvidence DeepConsult	AI agent synthesizing research studies to guide drug discovery and clinical decisions.	2025
Personalized medicine and treatment planning	Tempus	AI-driven precision oncology platform analyzing clinical and genomic data.	2015
	Flatiron Health	AI-powered oncology data platform for treatment planning.	2012
	Paige. AI	AI for cancer diagnosis and pathology image analysis.	2018
	Freenome	AI-based early cancer detection using blood-based biomarkers.	2014
	Owkin	AI federated learning for oncology research and biomarker discovery.	2016
	GRAIL	AI-powered liquid biopsy for multi-cancer early detection.	2016
	MedOrch	Multi-tool AI reasoning framework for diagnosis across multiple disease domains.	2025
	CareYaya MedaCareLLM + Smart Glasses	Personalized dementia care with facial/object recognition and reminders.	2024
Patient management	Babylon Health	AI chatbot for symptom checking and telemedicine.	2013
	Ada Health	AI-powered symptom checker and diagnosis assistant.	2016
	Buoy Health	AI-based triage system for guiding patients on next medical steps.	2017
	Woebot	AI chatbot for mental health support.	2017
	K Health	AI-powered primary care and symptom triage platform	2016
	CarePredict	AI wearable for monitoring elderly health and predicting falls.	2013
	Heidi Health	AI scribe generating structured documentation, summaries, and EHR integration.	2024–2025
	Eko AI-powered Stethoscope	AI diagnosis of heart failure, valve disease, arrhythmias within 15 s.	2025
Hospital workflow and administration	Olive AI (operations scaled back 2023)	AI-driven automation of hospital administrative tasks.	2017
	Nuance DAX (Dragon Ambient eXperience)	AI-powered medical transcription and clinical documentation.	2020
	Moxi	AI robotic assistant for hospital logistics and patient support.	2018
	Microsoft Dragon Copilot	AI assistant for note generation, summaries, referrals (EHR-integrated).	2025
Wearable and remote monitoring	Apple Health AI	AI-based health tracking (ECG, heart rate monitoring).	2018
	Fitbit AI	AI-driven health tracking and early disease detection.	2019
	Eko AI	AI-powered heart sound analysis for detecting cardiovascular diseases.	2015
	Biofourmis	AI for real-time remote patient monitoring and predictive analytics.	2015
	Withings Health Solutions	AI-based remote monitoring for hypertension, sleep apnea.	2021

#### Quantum computing in precision medicine

Quantum computing holds immense potential in overcoming computational challenges in precision medicine. Unlike classical computers, quantum systems leverage superposition and entanglement to execute high-dimensional analyses at unprecedented speeds. Quantum-assisted drug discovery is expected to revolutionize molecular simulations, improving the identification of optimal drug candidates (Danishuddin et al., 2025). Algorithms such as the Variational Quantum Eigensolver (VQE) enhance molecular interaction predictions, streamlining structure-based drug design. Additionally, quantum machine learning (QML) techniques are being applied to genomic analysis, improving biomarker identification and patient stratification. Quantum-enhanced deep learning models also hold promise in accelerating AI model training, leading to faster and more accurate clinical decision-making.

#### AI-driven industry-academia collaborations

Synergistic collaborations between academia and industry are critical for translating AI-driven healthcare innovations into practical applications. These partnerships facilitate translational research, ensuring the development of clinically validated and regulatory-compliant AI models. AI is increasingly being integrated into clinical trial design, optimizing patient recruitment and predictive modeling. AI-powered adaptive trials, leveraging Bayesian optimization techniques, enhance efficiency and reduce costs. Additionally, federated learning is enabling decentralized AI training, ensuring data privacy and regulatory compliance while facilitating multi-institutional collaborations. AI-powered digital twins virtual patient models simulating disease progression and treatment response are revolutionizing personalized medicine. These models are instrumental in optimizing therapeutic interventions, particularly in complex diseases such as cancer and rare genetic disorders. Industry-academia partnerships are driving the adoption of digital twin technology, further advancing precision medicine.

## Conclusion

This article has delineated the pivotal role of artificial intelligence (AI) in the evolution of personalized medicine, underscoring its capacity to significantly enhance diagnostic precision, optimize therapeutic strategies, and predict patient outcomes with remarkable accuracy. Key insights from our discussion highlight AI’s transformative potential in the realm of healthcare, particularly through advanced machine learning (ML) algorithms, deep learning frameworks, and the integration of vast, multidimensional datasets. AI facilitates the identification of complex biomarkers, the development of predictive models, and the refinement of treatment protocols, thus enabling precision medicine that is tailored to the individual patient’s unique genetic, environmental, and clinical characteristics. AI’s integration into precision medicine is poised to revolutionize several domains, including genomics, drug discovery, and imaging. In genomics, AI-driven tools can parse large-scale genomic data to identify novel genetic variants, predict disease risk, and guide the design of targeted therapies. In drug discovery, AI accelerates the identification of new therapeutic compounds by simulating molecular interactions and predicting their efficacy. Moreover, in clinical imaging, AI models are already enhancing diagnostic accuracy by automating image analysis, detecting anomalies with greater sensitivity than traditional methods. Despite these advances, substantial challenges persist in the full-scale implementation of AI in clinical practice. These include data heterogeneity, the need for robust validation frameworks, regulatory considerations, and ensuring patient data privacy. Furthermore, the clinical integration of AI must account for the broader healthcare ecosystem, where scalability and cost-effectiveness remain significant barriers. Addressing these challenges will require multidisciplinary efforts to develop standardized methodologies for AI deployment, ensure transparency in AI-driven decision-making, and foster collaboration between AI researchers and healthcare providers to ensure these technologies are clinically relevant and accessible. Future research should focus on optimizing the accuracy and interpretability of AI algorithms, particularly in terms of their ability to generalize across diverse populations and clinical conditions. Advancements in explainable AI (XAI) will be crucial for clinicians to understand the rationale behind AI-driven recommendations, fostering trust and enhancing clinical decision-making. Furthermore, efforts to harmonize data across disparate platforms and ensure interoperability between AI systems and existing healthcare infrastructures will be vital to achieving seamless integration. In summary, AI represents a paradigm shift in personalized medicine, with the potential to redefine healthcare delivery by offering highly individualized, data-driven treatments. As the field matures, it will be essential to navigate both the technological and ethical challenges that accompany AI adoption. With sustained research and collaboration, the future of personalized medicine is set to evolve into an era characterized by more precise, effective, and equitable healthcare for all.
